# Gastric inverted polyp mimicking submucosal tumor

**DOI:** 10.1055/a-2776-5368

**Published:** 2026-01-23

**Authors:** Cuimei Ma, Zongjing Hu, Shuqing Zhu, Dehuai Jing

**Affiliations:** 1 562122 Department of Gastroenterology, Affiliated Hospital of Jining Medical University, Jining, China; 2 562122 Endoscopy Department, Affiliated Hospital of Jining Medical University, Jining, China

**Fig. 1:**
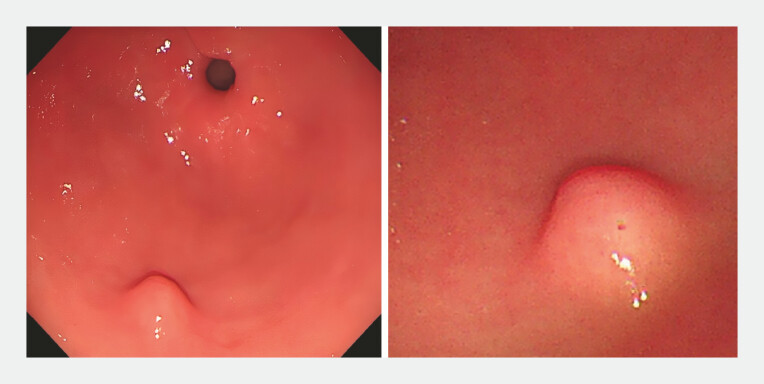
Upper gastrointestinal endoscopy revealed a submucosal tumor in the greater curvature of the gastric antrum with a smooth surface and a pinpoint depression at its apex.

**Fig. 2:**
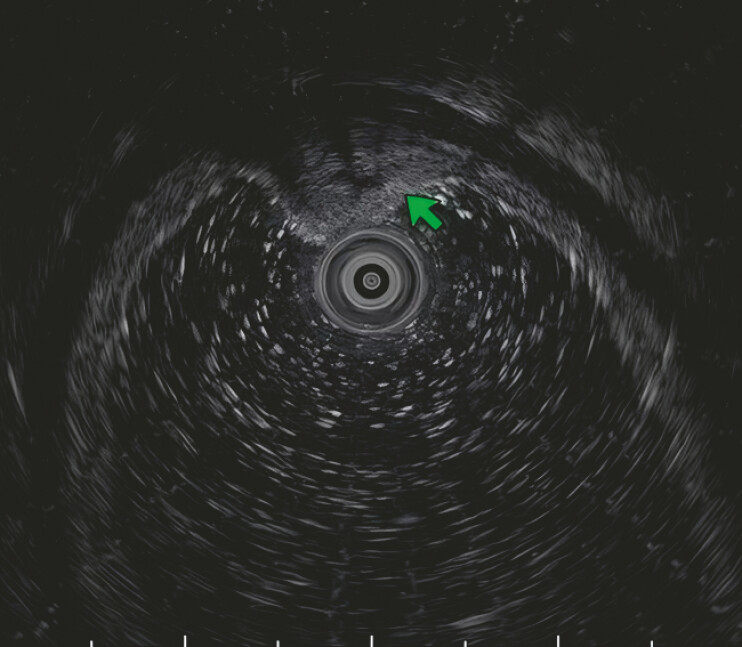
Endoscopic ultrasonography revealed a heterogeneous medium-to-high echogenicity with anechoic structures visible internally, originating from the submucosal layer.

**Fig. 3:**
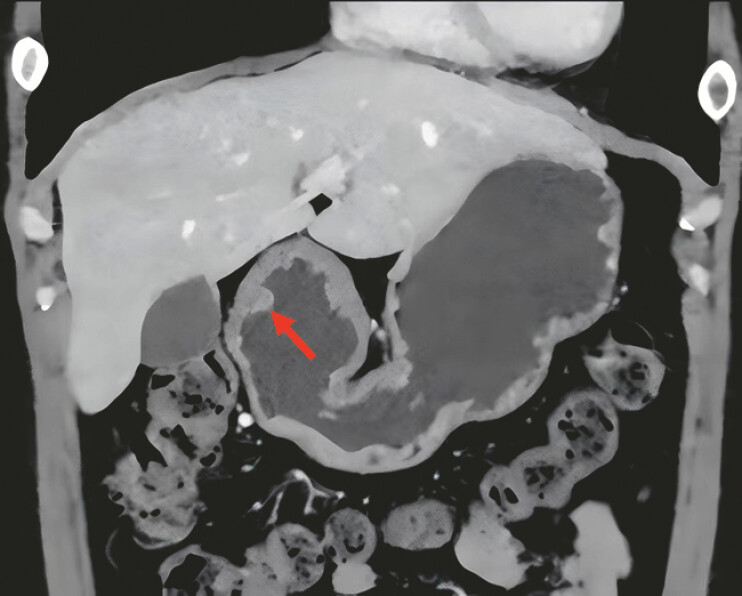
Enhanced computed tomography revealed the presence of a high density lesion in the gastric antrum (arrows).

**Fig. 4:**
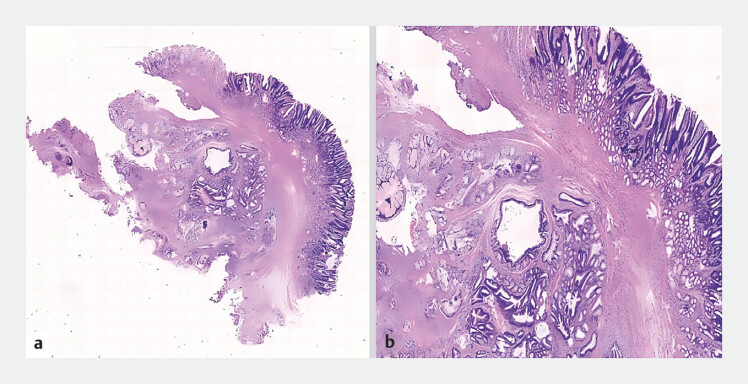
**a** A HE-stained image (×4) showed an inverted growth manner, within the submucosal layer, composed of variably sized and shaped dilated glands surrounded by smooth muscle bundles. **b** A HE-stained image (×10) showed lobulated mucous neck glands without cellular atypia are observed, with some glands showing cystic dilation. HE, hematoxylin and eosin.

A 38-year-old woman presented with a submucosal tumor in the gastric antrum. The patient reported no significant discomfort. Upper gastrointestinal endoscopy revealed a submucosal tumor in the greater curvature of the gastric antrum with a smooth surface and a pinpoint depression at its apex ( Fig. 1 ). Endoscopic ultrasonography revealed a heterogeneous medium-to-high echogenicity with anechoic structures visible internally, originating from the submucosal layer ( Fig. 2 ). Enhanced computed tomography revealed the presence of a high density lesion in the gastric antrum (arrows; Fig. 3 ). To further characterize the lesion, we performed endoscopic submucosal dissection (ESD) using a hook knife ( Video 1 ). Complete resection of the tumor was achieved. Pathology showed an inverted growth manner, composed of variably sized and shaped dilated glands surrounded by smooth muscle bundles, with acellular atypia and some exhibiting cystic dilatation ( Fig. 4 ). Therefore, the diagnosis of gastric inverted polyp (GIP) was made. The patient was placed on a 24-hour fasting regimen postoperatively and administered a proton pump inhibitor. The patient recovered well and was discharged 3 days after the operation. Follow-up 2 weeks after operation, the patient reported no significant discomfort.

Endoscopic submucosal dissection of gastric inverted polyp mimicking a submucosal tumor.Video 1

GIP is a rare polyp with an inverted submucosal growth trend, accounting for <1% of all gastric polyps 1 . GIP often presents as SMT-like lesions covering normal gastric mucosa, and is easily confused with true submucosal tumors 2 . When encountering similar cases, GIP should be included as one of the options in the differential diagnosis list. GIP has been reported that it is often associated with gastric adenocarcinoma or gastric mucosal epithelial dysplasia 3 . The diagnosis mainly relies on pathological diagnosis. Diagnostic resection is currently used in many cases of GIP. Our experience suggests that ESD is a safe and effective method for both the diagnosis and treatment of GIP.

Endoscopy_UCTN_Code_CCL_1AB_2AD_3AB
